# Lightweight Spatial-Frequency Collaborative Interaction Network for RGB-D Salient Object Detection

**DOI:** 10.3390/s26123708

**Published:** 2026-06-10

**Authors:** Yitong Lu, Ziguan Cui

**Affiliations:** 1 Portland Institute, Nanjing University of Posts and Telecommunications, Nanjing 210023, China; p23000617@njupt.edu.cn; 2 College of Telecommunications and Information Engineering, Nanjing University of Posts and Telecommunications, Nanjing 210003, China

**Keywords:** salient object detection, lightweight, frequency domain, RGB-D images

## Abstract

RGB-D salient object detection (SOD) aims to segment the most prominent objects from the background with a pair of given RGB and depth images. Existing RGB-D methods usually rely on heavy backbones to achieve high accuracy, while current lightweight methods struggle to maintain competitive performance. To break this intractable trade-off between effectiveness and model complexity, we propose a Lightweight Spatial-Frequency Collaborative Interaction Network (SFCINet), a unified and highly efficient framework. The core of SFCINet resides in the synergy between spatial-domain features and frequency-domain global priors. Specifically, we introduce the Spatial-Frequency Synergy (SFS) module, which shifts the perspective to a joint complex Fourier domain. By adaptively learning and optimizing the decoupled amplitude and phase components, it effectively isolates clutter to yield a purified global frequency-synergized prior, which modulates the spatial branches to eliminate cross-modal discrepancies for subsequent feature fusion while supplementing global information during decoding. To alleviate the interference caused by cross-modal representation discrepancies, we design the Cross-Guidance Interaction (CMGI) module, which employs a reciprocal anchoring mechanism. It guides the counterpart to mutually filter irrelevant noise and select task-relevant information, achieving fusion in an efficient manner. Finally, we present a Calibrated Hierarchical Decoder (CHD), which injects frequency-synergized global priors into the hierarchical decoding process. It re-establishes the connection between the frequency and spatial domains, ultimately achieving global-local consistency. Extensive experiments demonstrate that SFCINet delivers superior performance over state-of-the-art methods.

## 1. Introduction

Salient object detection (SOD) focuses on detecting most distinctive objects or regions within an image, which is a crucial task in computer vision. It simulates the human visual system (HVS) and segments the most prominent parts from the background, which benefits many applications, including quality assessment [1], autonomous driving [2], semantic segmentation [3], visual tracking [4], and video summarization [5].

In the past several years, RGB-D salient object detection methods have undergone rapid development, demonstrating their superior performance by utilizing depth cues to enhance the recognition and localization ability of SOD methods. They efficiently mitigated the challenges brought by low-texture contrast or complex background. Yi et al. [6] designed a novel method for RGB-D salient object detection, which achieves bidirectional selection between RGB and depth information. It fully exploits the rich textural information from RGB and the strong structural information embedded in depth. Wei et al. [7] proposed a PDNet to overcome the limitations of traditional RGB-based methods in complex background environments by leveraging the abundant spatial and positional information provided by depth images to enhance the separation of salient objects. Whereas these methods have shown outstanding performance in metrics, most of them rely on high computational cost and large backbones, which suffer from high deployment barriers due to their substantial computational overhead. Consequently, how to achieve the balance between efficiency and model complexity remains a major issue. Lightweight RGB-D SOD methods have been developed to address the problem by adopting and improving more efficient frameworks. Compared with heavyweight networks, their performance is unsatisfactory and struggles to handle complex scenes. This motivates us to explore complementary cues beyond the spatial domain to compensate for the limited representation capacity of lightweight architectures.

Specifically, frequency information has proven effective in capturing global contrast and structural boundaries. Traditional models based on early hand-crafted features successfully leveraged this characteristic, yielding remarkable results. For example, Achanta et al. [8] proposed a frequency-tuned approach that computes pixel-level saliency by measuring the color difference between the global mean image feature and Gaussian-blurred pixel vectors. It is simple to implement and is computationally efficient, which retains more boundary information. Ma et al. [9] introduced wavelet transforms into saliency analysis, decomposing images into multiple frequency sub-bands. It possesses the capacity to simultaneously extract local high-frequency edge details and global low-frequency spatial structures. However, these traditional models are inherently constrained by their reliance on hand-crafted features, even when incorporating frequency-domain analysis. Lacking high-level semantic guidance, the representation capacity of these hand-crafted features is insufficient to distinguish genuine salient objects from high-frequency background interference. With the rise of deep learning, researchers have begun integrating frequency analysis into neural architectures to enhance semantic representation. For instance, Jin et al. [10] proposed FCMNet, which utilizes frequency-aware attention to strengthen cross-modal interaction. It achieves synchronization of multi-modal features by highlighting shared salient components in the spectral domain. However, this approach typically employs frequency information only for global weighting and lacks a mechanism to distinguish modal-specific spectral discrepancies. Specifically, the inherent high-frequency noise in depth maps is often indiscriminately amplified during spectral interaction, leading to severe feature contamination. Consequently, achieving a purified and efficient spatial-frequency synergy remains a critical challenge.

To overcome the aforementioned limitations and achieve an effective spatial-frequency synergy, we identify several core bottlenecks that must be resolved. The first major challenge in lightweight RGB-D SOD hinges on the intrinsic conflict between global perception and computational complexity. Due to the limited receptive field of convolution, CNN-based architectures are incapable of effectively capturing global contextual features, failing to model the holistic contours and global structures of salient objects. Although Transformer-based models have alleviated the limitation by benefiting from self-attention mechanisms, their computational overhead remains prohibitively high for practical deployment. To break the bottleneck, we propose the Spatial-Frequency Synergy (SFS) module, shifting the perspective from the spatial domain to the frequency domain. By employing the Fast Fourier Transform (FFT), SFS maps the spatial features into a decoupled amplitude-phase spectral space, effectively capturing global signals in the spectral domain with much lower complexity. Unlike conventional uniform frequency processing, SFS exploits the distinct physical properties of these frequency components. In the Fourier spectral domain, the amplitude spectrum governs global energy distribution, macro-textures, and illumination styles, while the phase spectrum preserves the critical geometric skeletons, absolute object boundaries, and spatial topological structures. Depth maps lack fine texture and suffer from hole noise and artifacts on their high-frequency bands, while their low-frequency components hold macro-contours. Conversely, the high-frequency bands of RGB maps embrace sharp structural boundaries while their lower frequencies are heavily susceptible to deceptive semantic clutter and background distractions in complex scenes. By joint optimization in the complex frequency domain, the amplitude path adaptively acts as a targeted spectral filter to suppress the high-frequency noise contaminating the depth amplitude spectrum, thereby recovering the pristine global structural energy. The phase path leverages the discriminative RGB phase spectrum as an immutable geometric compass to structurally rectify and align the misaligned depth contours. Upon returning to the spatial domain, instead of natively fusing these features prematurely, SFS yields a purified global frequency-synergized prior alongside two enhanced single-modal spatial representations. This global prior is actively deployed to mutually modulate the spatial branches of both modalities, eliminating inherent feature discrepancies. Consequently, it ensures that the subsequent CMGI module can perform cross-modal feature fusion with enhanced efficiency and robustness across a structurally aligned spatial space. Concurrently, this prior is explicitly preserved to supplement macro-structural cues during the top-down decoding process.

The second challenge concerns the effective fusion of multi-modal features. It is well known that depth maps often contain substantial noise and exhibit low-texture characteristics. Direct fusion of depth and RGB features leads to feature contamination, significantly degrading the performance and robustness of the model. To handle this, we introduce the Cross-modal Guidance Interaction (CMGI) module. We adopt a reciprocal anchoring mechanism to constrain feature interaction. Utilizing the structurally consistent features refined by the SFS module, we derive a spatial confidence map for each modality. Rather than self-modulation, the spatial confidence derived from one modality acts as an immutable physical ‘anchor’ to explicitly constrain and regularize the valid feature activation space of another modality. The RGB branch enforces a gated suppression on the corrupted region where depth maps suffer from inherent noise. Meanwhile, where the RGB features inevitably encounter deceptive semantic clutter or foreground-background confusion, the invariant anchor from the depth branch forcefully constrains the RGB feature space, preventing its attention from drifting. This complementary bidirectional bounding enforces a topological interception of the information flow, blocking the modal-specific clutter from both sides.

Furthermore, to obtain high-quality saliency maps, we design the Calibrated Hierarchical Decoder (CHD) to integrate the global frequency-synergized prior from the SFS into the hierarchical decoding process. Different from standard decoders, we utilize these spectral priors as holistic geometric reference to govern the progressive feature reconstruction, preventing the structural drift during multi-scale feature fusions. Rather than merely recovering lost global context, this global-to-local guidance mechanism ensures that fine-grained spatial details are always regularized by the holistic structural consensus, forming the SFCINet into a unified framework where spectral-domain priors and spatial-domain features operate in organic synergy. Our main contributions can be summarized as follows:

1. We propose SFCINet, an efficient and unified framework for RGB-D SOD that enables spatial-frequency information interaction and synergistic fusion. By establishing a robust global-to-local guidance mechanism, it injects a global frequency-synergized prior into the decoding procedure, effectively mitigating structural drift during multi-scale feature fusions.
2. We present the SFS module, which shifts the perspective to the complex Fourier domain to capture holistic contextual features. By exploiting the distinct physical properties of amplitude and phase frequency spectral components, SFS suppresses depth noise while capturing sharp RGB structural boundaries, resolving the multi-modal spectral heterogeneity. It crystallizes a purified global frequency-synergized prior, and simultaneously calibrates the spatial features.
3. We introduce the CMGI module to achieve cross-modal fusion without contamination. Driven by a reciprocal anchoring mechanism, it utilizes the spatial confidence derived from the SFS-enhanced features as an invariant structural constraint to mutually gate both modalities, successfully blocking localized noise and deceptive semantic clutter.

## 2. Related Work

### 2.1. RGB Salient Object Detection

SOD for RGB images has been extensively studied for many years and existing methods can be divided into two main categories: traditional methods and deep learning methods. Traditional RGB SOD methods mainly rest on hand-crafted features, including boundary background, texture and so on. However, the constrained representation capacity of hand-crafted features restricts the performance of traditional SOD methods, resulting in the focus of SOD shifting towards sophisticated deep learning architectures. Early Convolutional Neural Network (CNN)-based SOD models show outstanding performance in extracting local textures. MENet [11] achieved impressive results by implementing multiple enhancement strategies across pixels, regions, and objects to refine feature representations. However, due to the limited receptive field of the convolution kernel, CNNs have insufficient ability to capture the global context features. Over the past few years, Vision Transformers (ViTs) have been employed to solve the problem. For example, VST [12] leveraged self-attention mechanisms for robust long-range dependency modeling, which excels at capturing global context. However, the significant computational cost of these heavyweight models often limits their practicality. Recently, various lightweight SOD methods have gradually emerged to strike a better balance between effectiveness and accuracy. For example, Wang et al. proposed LARNet [13], which introduces a brain-inspired context gating module to realize deep multi-level feature fusion at the global level. Wang et al. [14] presented an extremely lightweight wavelet neural network named ELWNet, which integrates wavelet transform modules (WTMs) and fusion modules (WTFMs) into a convolutional architecture. Although the aforementioned methodologies are primarily designed for single RGB images, they provide profound implications for RGB-D SOD research.

### 2.2. RGB-D Salient Object Detection

Distinct from RGB SOD [15], RGB-D SOD [16] methods integrate depth maps as a supplementary modality, leveraging spatial geometry and structural information to facilitate salient object detection. Modern high-performance models often employ dual-stream backbones, CNN or Transformer, to extract features independently before performing complex cross-modal feature fusion, which relies on large model size and high computational cost. For example, Wei et al. [17] utilized dual ResNet-50 backbones to explore the consistency and complementarity between modalities through a modal-aware interaction mechanism. Zhou et al. [18] developed IRFR-Net, which reshaped features recursively to refine saliency maps across multiple levels. To capture long-range dependencies, Liu et al. proposed VST [12], a pure Transformer-based framework that leverages multi-level Transformer blocks to unify feature extraction and fusion. Furthermore, Wang et al. [13] presented an adaptive fusion bank for multi-modal SOD, which dynamically coordinated diverse fusion strategies to enhance the robustness of feature integration. Though these methods demonstrated remarkable accuracy, their architectures often face two fundamental bottlenecks. First, capturing global contextual dependencies via spatial self-attention intrinsically incurs significant parameter redundancy and high computational latency, posing insurmountable latency challenges for real-time inference on resource-constrained devices. Second, most existing fusion mechanisms ignore the inherent information discrepancy between the two modalities. When conducting spatial alignments, the inherent high-frequency noise and structural inaccuracies in depth maps easily act as misleading guidance. This false induction misdirects the RGB branch to extract erroneous features, inevitably leading to severe cross-modal contamination.

### 2.3. Lightweight RGB-D Salient Object Detection

The majority of RGB-D SOD methods employ heavyweight architectures, such as ResNet101, Swin Transformer, and ViT, to achieve higher accuracy, posing a heavy burden on resource-constrained edge devices. To address this challenge, lightweight RGB-D SOD has emerged as a critical research direction. These methods replace heavyweight encoders with efficient backbones like MobileNetV2 [19], ShuffleNet [20], or MobileViT [21]. For instance, Jin et al. proposed MoADNet [22], which introduces a mobile asymmetric dual-stream encoder to minimize computational redundancy. By assigning disparate computational budgets to the RGB and depth branches based on their information density, MoADNet preserves essential modal features while significantly lowering the overall FLOPs compared to conventional symmetric dual-stream counterparts. Shifting away from the traditional dual-branch paradigm, Zhang et al. [23] designed a robust single-stream architecture. This approach streamlines the feature extraction and fusion process into a unified pipeline, effectively eliminating the parameter redundancy inherent in parallel encoders and achieving high-speed real-time inference on resource-constrained edge devices. Furthermore, Zhang et al. [24] introduced the MCCNet. It leverages a suite of cross-modal complementation modules to exploit the fine-grained, reciprocal interactions between RGB and depth cues, refining saliency maps in a computationally efficient manner. These methodologies underscore that through meticulous structural optimization and targeted feature interaction, lightweight models can achieve competitive performance while remaining viable for industrial deployment.

### 2.4. Applications of Frequency Information

Recently, the exploration of frequency information has witnessed remarkable advancements in image processing. It shifts the perspective from the spatial domain to the frequency domain, endowing the model with excellent global awareness. Unlike spatial convolutions that are restricted by local receptive fields, the Fast Fourier Transform (FFT) allows models to capture long-range dependencies across the entire image in the frequency domain. In contrast to the self-attention mechanism, FFT has a much lower computational complexity of O(NlogN) instead of $O(N^{2})$. Driven by this efficiency, several efforts have been made to integrate spectral analysis into deep learning frameworks. For instance, DeepRFT [25] designs Fourier convolution-based residual blocks to capture multi-level frequency information, effectively expanding the receptive field for high-quality image deblurring. Qiao et al. [26] proposed a Fourier-based framework for unpaired image restoration, which learns depth-density priors within the spectral domain to effectively recover structural details and suppress complex degradations. In specific detection tasks, Zhao et al. [27] highlighted that objects and backgrounds with high spatial similarity become more discriminative when processed in the frequency domain. Furthermore, for multi-modal scenarios, Zhou et al. [28] introduced a lightweight framework for RGB-Thermal SOD that jointly mines spatial, channel, and frequency-domain cues, validating the viability of frequency-aware mining for resource-constrained multi-modal tasks. FCMNet [10] extends the frequency channel attention mechanism into the RGB-D pipeline by computing 2D DCT coefficients independently for each single-modal stream. However, these existing multi-modal frequency methods treat the frequency spectrum as a unified whole, focusing on capturing and aggregating information across isolated high- or low-frequency bands. Different image modalities exhibit entirely different physical properties in their respective high- and low-frequency components, treating the spectrum as a uniform whole inevitably poses a severe risk of cross-modal feature contamination. Specifically, in RGB-D tasks, the high-frequency band of the depth stream is heavily dominated by localized noise and measurement artifacts, whereas the high-frequency band of the RGB stream contains pristine, highly discriminative object boundaries. Without a joint spectral space to coordinate the cross-modal consensus, these frameworks cannot decouple the intertwined modal anomalies, inevitably causing severe noise or deceptive background clutter to propagate into the subsequent multi-modal fusion.

To overcome these limitations, our proposed SFS module executes a targeted amplitude-phase separation to adaptively optimize these fundamental frequency spectral components. Crucially, instead of treating the refined results as isolated descriptive features, we formulate them as a robust global frequency-synergized prior that governs twofold core functions. On one hand, this global prior is actively deployed to mutually modulate the spatial representations of both modalities instead of fusing with them. It aims to derive a reliable cross-modal consensus, thereby seamlessly guiding and enabling the subsequent CMGI module to execute a far more optimal and robust feature fusion. On the other hand, it concurrently acts as an overarching global prior directly injected into the subsequent Decoder stage to explicitly supplement the structural consensus during top-down feature reconstruction. By explicitly partitioning and purifying these components, the SFS effectively isolates single-modal clutter while faithfully retaining the multi-modal structural consensus and global frequency-synergized priors.

## 3. Proposed Method

In this section, we first elaborate on the overall architecture of SFCINet. Then, we introduce the significant modules successively, including the SFS module, the CMGI module, and the CHD. Ultimately, we provide the loss functions.

### 3.1. Overall Architecture

**Figure 1:**
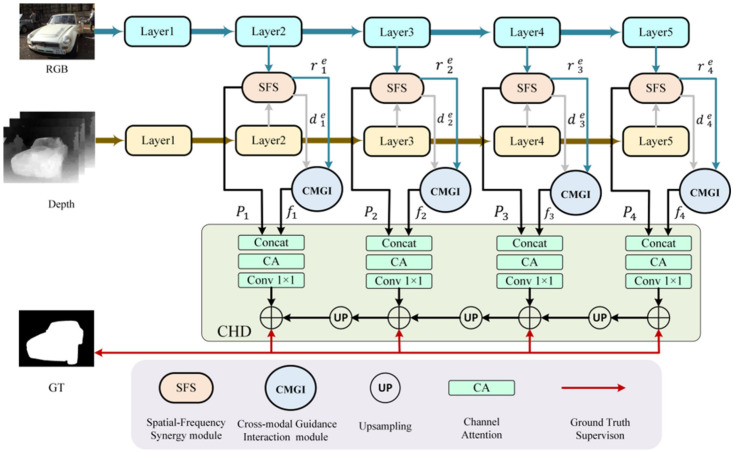
The architecture of the proposed SFCINet.

As shown in Figure 1, the overall architecture of SFCINet follows an encoder-decoder design, which is mainly composed of four components: a dual-branch encoder, SFS, CMGI, and CHD. The depth images are copied into three channels to match the dimensions of RGB images. We employ MobileViT as the backbone to extract multi-scale features from RGB and depth images, denoted as $r_{i}\;\left\{i=1,2,3,4\right\}$ and $d_{i}\;\left\{i=1,2,3,4\right\}$. These features are fed into the SFS module in pairs, obtaining frequency-aware refined spatial features $r_{i}^{e}$ and $d_{i}^{e}$, as well as a global frequency-synergized prior Prior, enhancing and calibrating features under the coordination of the frequency-spatial domain. Later, they flow into the CMGI module, resulting in the fused feature $f_{i}$. Here, they perform reciprocal guidance to purify these features by autonomously selecting and suppressing the characteristics of the other modality. By interacting on these already-purified features instead of raw inputs, CMGI successfully avoids spreading single-modal noise into the other stream. Finally, CHD is employed to integrate the multi-level fused features and the global frequency-synergized prior $P_{i}$, which acts as a reliable global layout to correct any remaining structural errors during the hierarchical decoding process, ultimately achieving global-local consistency.

### 3.2. Spatial-Frequency Synergy (SFS) Module

**Figure 2:**
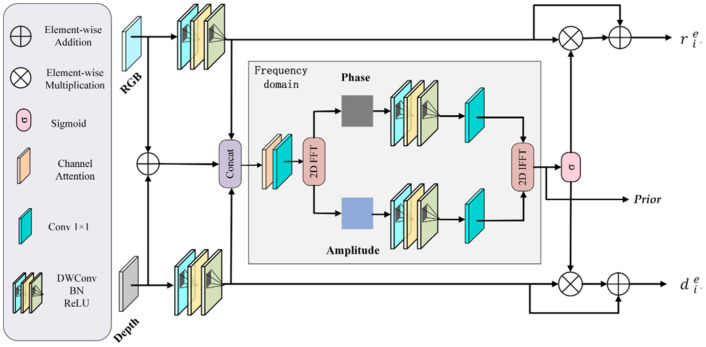
Structure of the Spatial-Frequency Synergy (SFS) module.

The traditional convolutional receptive field is limited, while the self-attention mechanism relies on a large amount of computational power, which is not conducive to the implementation of lightweight networks. To capture global contextual dependencies efficiently, the SFS module maps the spatial features into the frequency domain. As shown in Figure 2, first, we fuse the $r_{i}\;\left\{i=1,2,3,4\right\}$ and $d_{i}\;\left\{i=1,2,3,4\right\}$ to perform a cross-modal representation $f_{i}^{cat}\left\{i=1,2,3,4\right\}$, and the Channel Attention operator is used to optimize the concatenated channels. Then, we apply the 2D Fast Fourier Transform (FFT) to transform it from the spatial domain to the frequency domain, which can be formulated as follows:

(1) $$f_{i}^{cat}=Cat\left(r_{i}+d_{i},\;r_{i},\;d_{i}\right),$$

(2) $$f_{i}^{\prime }={Conv}_{1\times 1}(CA(f_{i}^{cat})⊗f_{i}^{cat}),$$

(3) $$A,\;P=FFT(f_{i}^{\prime }),$$

where Cat(·) denotes concatenation, and ${Conv}_{1\times 1}(\cdot )$ represents 1 × 1 convolution. And FFT(·) denotes 2D Fast Fourier Transform, which generates amplitude and phase of $f_{i}^{\prime }$ in the frequency domain. A represents the amplitude, and P represents the phase.

Afterward, amplitude and phase information is learned and optimized respectively in dual-path networks, generating a global frequency-synergized prior Prior. Prior is used for decoding, and it also generates a set of gated weights $\left\{m_{r},m_{d}\right\}$ through convolutional learning. The above process can be expressed as follows:

(4) $$A_{e}={DWConv}_{3\times 3}(BN(ReLU(A))),$$

(5) $$P_{e}={DWConv}_{3\times 3}(BN(ReLU(P))),$$

(6) $$Prior=IFFT(A_{e},P_{e}),$$

(7) $$\{m_{r},m_{d}\}=\sigma ({DWConv}_{3\times 3}(BN(ReLU(Prior)))),$$

where ${DWConv}_{3\times 3}(\cdot )$ denotes 3×3 Depthwise Convolution; BN(·) denotes Batch Normalization; ReLU(·) represents Rectified Linear Unit; IFFT(·) denotes 2D Inverse Fast Fourier Transform; σ represents Sigmoid Activation Function.

The raw inputs also undergo convolutional learning and a gated mechanism, modulating the spatial features. A residual connection is also employed to enhance the representation. The above process reconciles spatial textures with global frequency layouts, obtaining the enhanced and calibrated features, which can be formulated as follows:

(8) $$r_{i}^{\prime }={DWConv}_{3\times 3}(BN(ReLU(r_{i}))),$$

(9) $$d_{i}^{\prime }={DWConv}_{3\times 3}(BN(ReLU(d_{i}))),$$

(10) $$r_{i}^{e}=r_{i}^{\prime }⊗m_{r}+r_{i}^{\prime },$$

(11) $$d_{i}^{e}=d_{i}^{\prime }⊗m_{d}+d_{i}^{\prime }.$$

The SFS module serves as a spectral-spatial bridge that reconciles local modality features with global structural consensus. By enriching the spatial features with frequency-domain characteristics, it alleviates discrepancy gap in the subsequent cross-modal interaction.

### 3.3. Cross-Modal Guidance Interaction (CMGI) Module

**Figure 3:**
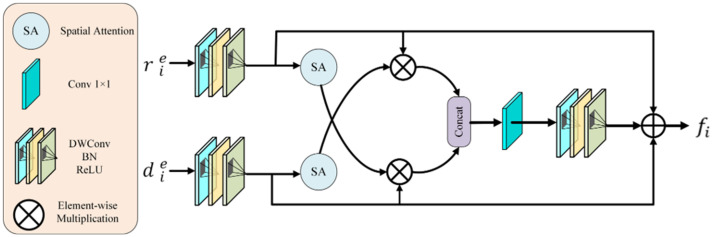
Structure of the Cross-modal Guidance Interaction (CMGI) module.

Given the characteristics of depth maps and RGB images, for example, the depth map contains a lot of noise, while the RGB image has a messy background. A simple addition or concatenation often leads to feature contamination and loss. To alleviate these problems, we design the CMGI, as illustrated in Figure 3, which utilizes the spatial confidence of one modality to anchor the other, ensuring the efficient transmission of information.

First, processed features $r_{i}^{e}$ and $d_{i}^{e}$ are learned by a set of convolutions. Then, we introduce a spatial attention (SA) mechanism to derive the spatial confidence maps $\left\{M_{r},\;M_{d}\right\}$. Then, the RGB and Depth branch are respectively constrained by each other, in order to reach a consensus on features. The process can be expressed as follows:

(12) $$R_{i}^{e}={DWConv}_{3\times 3}(BN(ReLU({r\;}_{i}^{e}))),$$

(13) $$D_{i}^{e}={DWConv}_{3\times 3}(BN(ReLU({d\;}_{i}^{e}))),$$

(14) $$M_{r}=SA(R_{i}^{e}),$$

(15) $$M_{d}=SA(D_{i}^{e}),$$

(16) $$R_{i}^{D}=M_{r}⊗R_{i}^{e},$$

(17) $$D_{i}^{R}=M_{d}⊗D_{i}^{e},$$

where SA(·) denotes the spatial attention mechanism.

After being concatenated, it continues to undergo compression and learning, and finally connects with the residual connection of its own branch, thereby enhancing the feature representation and stability, which can be formulated as follows:

(18) $$F_{i}={Conv}_{1\times 1}(Cat(R_{i}^{D},\;D_{i}^{R})),$$

(19) $$F_{i}^{\prime }={DWConv}_{3\times 3}(BN(ReLU(F_{i}))),$$

(20) $$f_{i}=F_{i}^{\prime }+R_{i}^{e}+D_{i}^{e}.$$

Through cross-modal transmission and optimization of information, CMGI provides the decoder with consensus-driven local details. This ensures that the decoder can focus purely on top-down decoding with the global spectral priors for final saliency restoration.

### 3.4. Calibrated Hierarchical Decoder (CHD)

The CHD is responsible for the progressive integration of the global frequency-synergized prior $P_{i}$ and the interactive feature $f_{i}$. To prevent the accumulation of redundant information and alleviate the semantic gap during upsampling and concatenation, we introduce channel attention to perform feature compression and dimensionality reduction. Subsequently, the results undergo channel reduction and connect to the lower level, which can be expressed as follows:

(21) $$f^{i}=Cat(f_{i},{Prior}^{i}),$$

(22) $$f_{ca}^{i}={Conv}_{1\times 1}(f^{i}⊗CA(f^{i})),$$

(23) $$f_{final}^{i}=f_{ca}^{i}+Up(f_{final}^{i+1}),$$

where Up(·) denotes bilinear interpolation. This skip-connection-style integration allows the network to effectively propagate saliency information across different resolutions while maintaining structural integrity.

By anchoring the progressive decoding process with the global frequency-synergized prior, the network ensures that global frequency insights and local spatial precision operate in organic synergy, forming a unified SFCINet framework.

### 3.5. Loss Function

In the training phase, we adopt a hybrid loss consisting of the BCE loss and the Dice loss, a multi-scale supervision strategy to supervise the $f_{final}^{k}\left\{k=1,2,3,4\right\}$, ensuring the generation of high-quality saliency maps.

The BCE loss is widely adopted for pixel-wise classification, independently evaluating the discrepancy between the predicted probability and the ground truth at each pixel. For the BCE loss at the k-th level, it can be formulated as follows:

(24) $$L_{BCE}\left(G_{s}^{k},P^{k}\right)=-\sum_{i=1}^{H}\sum_{j=1}^{W}\left[G_{s}^{k}\left(i,j\right)\log\left(P^{k}\left(i,j\right)\right)+\left(1-G_{s}^{k}\left(i,j\right)\right)\log\left(1-P^{k}\left(i,j\right)\right)\right].$$

However, calculating the loss pixel by pixel often neglects the global structural integrity of the salient objects. To alleviate this, we introduce the Dice loss, which directly measures the overlap similarity between the predicted region and the ground truth at the image level, thereby maintaining the overall consistency of the objects. The Dice loss is formulated as follows:

(25) $$L_{\mathrm{Dice}}\;\left(G_{s}^{k},P^{k}\right)=1-\frac{2\sum_{i,j}P^{k}\left(i,j\right)G_{s}^{k}\left(i,j\right)}{\sum_{i,j}P^{k}\left(i,j\right)+\sum_{i,j}G_{s}^{k}\left(i,j\right)}.$$

Therefore, the overall loss is obtained:

(26) $$L_{\mathrm{sal}}=\sum_{k=1}^{4}\left[\left(G_{s}^{k},P^{k}\right)+L_{\mathrm{Dice}}\;\left(G_{s}^{k},P^{k}\right)\right],$$

where H and W denote the height and width of the image; $\left(i,j\right)$ denotes the pixel coordinate; $P^{k}\left(i,j\right)$ and $G_{s}^{k}\left(i,j\right)$ denote the predicted saliency probability and the ground truth label, respectively, at the k-th level.

## 4. Experimental Results and Analysis

### 4.1. Datasets

We select five mainstream RGB-D datasets for experiments: SIP [29] (929 image pairs), NJU2K [30] (1985 image pairs), NLPR [31] (1000 image pairs), STERE [32] (1000 image pairs), and DUT-RGBD [33] (1200 image pairs). SIP is a human-centric dataset which focuses on salient person detection in outdoor environments. The depth maps were captured using the dual-camera sensor system of a Huawei Mate 10 smartphone, sourced from Huawei Technologies Co., Ltd. (Shenzhen, China). NJU2K, collected from the Internet and 3D movies, contains diverse indoor and outdoor scenes. The depth maps in this dataset were acquired via the Fujifilm FinePix Real 3D W3 camera sensor, sourced from Fujifilm Holdings Corporation (Tokyo, Japan), or extracted directly from 3D movies to obtain diverse spatial structures. NLPR is captured by a Microsoft Kinect sensor, which was sourced from Microsoft Corporation (Redmond, WA, USA), including various objects under different lighting conditions. STERE focuses on multiple persons in complex poses and real-world occlusions. For this dataset, depth maps were generated through computational stereo matching algorithms rather than direct sensor capture, specifically targeting complex and unconstrained in-the-wild scenarios. DUT-RGBD involves multiple salient objects, low-contrast foregrounds, and complex background clutter. The depth maps were captured with a Lytro light field camera sensor, sourced from Lytro, Inc. (Mountain View, CA, USA), to effectively handle transparent objects and intricate structures. For fair comparison, the training datasets are selected from NJU2K, NLPR, and DUT-RGBD, which include 1485, 700, and 800 image pairs, respectively, while the remaining images are used for testing and evaluation. These training and testing splits fully and strictly follow the publicly available standard partitions universally adopted in the RGB-D SOD community.

### 4.2. Evaluation Metrics

For fairness and comprehensive comparison, we adopt five widely accepted metrics, including Precision-Recall (PR), F-measure ($F_{\beta }$) [8], mean absolute error (M) [34], S-measure (S) [35], and E-measure (E) [36]. PR curves plot the precision against recall, presenting a comprehensive visualization of the model’s performance. $F_{\beta }$ provides a balanced evaluation of the performance in balancing precision and recall. M quantifies the average pixel-wise discrepancy between the predicted saliency map and the ground truth. S focuses on the structural similarity, while E reflects enhanced alignment and global foreground consistency. They jointly conduct a comprehensive assessment of the model’s performance.

### 4.3. Implementation Details

We adopt MobileViT as our dual-branch backbone, which is initialized with official weights pre-trained on ImageNet, and remove other operations after layer 5. Our SFCINet is trained and inferred effectively on a single NVIDIA RTX 4060 GPU (8 GB VRAM; NVIDIA Corporation, Santa Clara, CA, USA). The input RGB and depth images are resized to 256×256 during the training and testing process. The SFCINet is trained for 200 epochs, with an initial learning rate of 1 × 10^−4^, and a weight decay of 0.1 every 50 epochs. In addition, the batch size is set to 8. To boost the robustness of our model, we adopt mainstream data augmentation strategies such as rotation, image flipping, and random rotation during the data processing.

### 4.4. Performance Comparison

To comprehensively evaluate the performance of SFCINet, we select 16 state-of-the-art methods for comparison. These methods are categorized into two groups: (1) heavyweight models, shown in Table 1, including CDNet [37], CCAFNet [38], CAVER [39], MPDNet [40], AMINet [41], HiDANet [42], TPCL [43], and DMGNet [44]; (2) lightweight models, shown in Table 2, including MoADN [22], MMF [45], LSNet [46], AirSOD [47], HENet [48], MAGNet [49], FasterSal [23], and BTNet [50].

(1) Quantitative Comparison

Compared with heavyweight models, as shown in Table 1, SFCINet demonstrates superior performance across most benchmarks. Notably, our model achieves the highest F-measure and E-measure on the SIP, NJU2K, NLPR, and STERE datasets, consistently outperforming models with significantly larger parameter scales. We perform best on F-measure and E-measure, especially that we make great progress, increasing $F_{\beta }$ from 0.892 to 0.911 on the highly challenging SIP dataset. In contrast to those heavyweight models, our proposed method enjoys significantly fewer parameters and much lower computational complexity (FLOPs), while delivering better overall performance. For example, compared to heavyweight competitors like TPCL (129.47M Params, 212.02G FLOPs), our network contains only 10.08M parameters and 4.27G FLOPs, which reduces the parameter size and computational burden by over 90%. But we outperform the competitors in terms of F-measure by approximately 1% on average. This fully demonstrates its remarkable model efficiency and great potential for practical deployment.

Compared with lightweight models, Table 2 presents the results against eight advanced lightweight methods. SFCINet ranks first across all metrics on the SIP and DUT-RGBD datasets, and achieves the highest F-measure and E-measure on all datasets. Instead of concatenating depth and RGB images and processing them via a unified shared backbone, our method leverages two separate backbones to model RGB and depth features, respectively. Consequently, our model is not competitive in parameters and FLOPs against some state-of-the-art lightweight models, but it achieves considerable performance gain benefiting from the efficient processing of cross-modal information via the dual-backbone network. For instance, we achieve a relative improvement of approximately 6.55% compared with AirSOD on the SIP although it is lighter in terms of parameters. It strongly proves that the SFCINet achieves a desirable trade-off between efficiency and accuracy.

**Figure 4:**
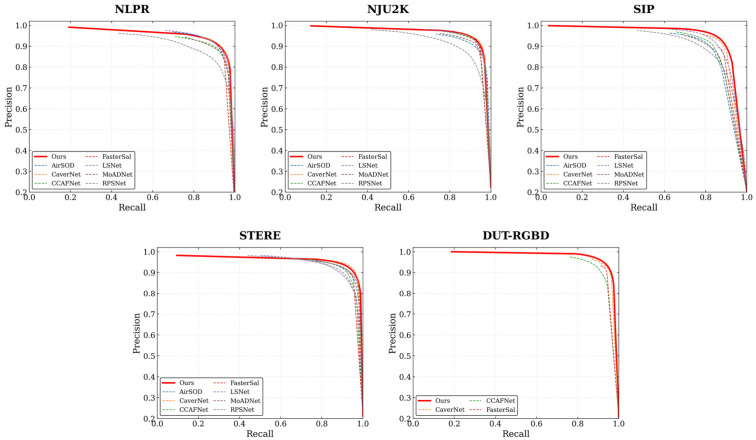
PR curves of the proposed method and state-of-the-art methods on five datasets.

Additionally, we pose the Precision-Recall (PR) curves across the five benchmark datasets, as illustrated in Figure 4. It can be intuitively observed that our PR curves stay above those of other methods, demonstrating its outstanding performance.

(2) Qualitative Comparison

**Figure 5:**
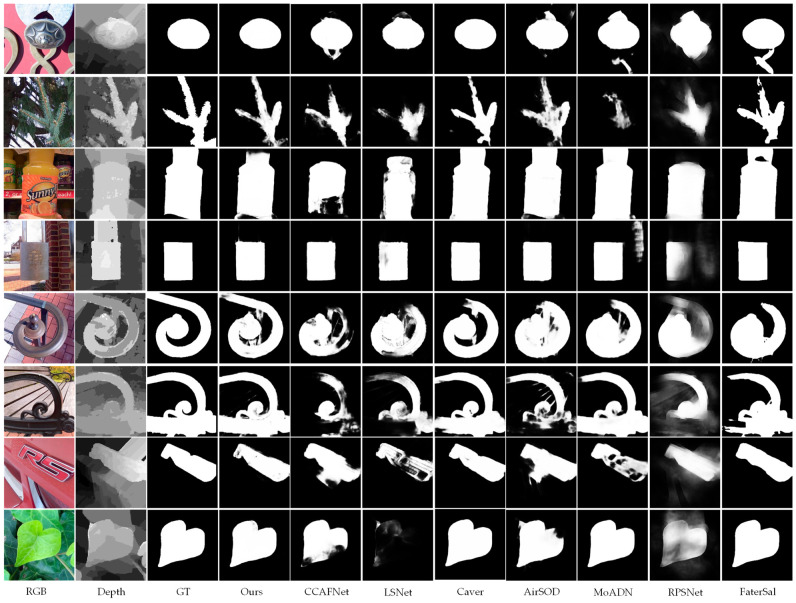
Qualitative comparisons with state-of-the-art methods.

For a qualitative comparison, we select several representative detection results, as illustrated in Figure 5. These examples contain several challenging scenarios, including situations with complex backgrounds (rows 1–2), poor depth maps (rows 3–4), intricate structures (rows 5–6), and low contrast (rows 7–8). Specifically, in rows 1–2, the patterns behind the door ring and the foliage beneath the pine needles create a complex background distraction. Our SFCINet effectively suppresses complex background clutter and extracts fine structures, while others often suffer from background confusion, leading to blurred boundaries and false detections. In rows 3–4, poor depth maps have significant noise and low contrast, causing significant misguidance for RGB features. Our SFCINet eliminates the distracting information and maintains the integrity and clear boundaries of the salient objects. In rows 5–6, depicting a spiral handrail and a bench armrest with intricate topologies, our model sharply preserves the internal hollow regions, while other methods merge these details into blurred blobs.

Finally, in rows 7–8, the car logo and green leaves have similar colors to their respective backgrounds, the SFCINet successfully distinguishes salient objects under extreme low-contrast conditions, while other models result in structural disintegration and object fragmentation. These visual results clearly demonstrate that our proposed method can more precisely locate salient objects across various extreme scenarios.

### 4.5. Ablation Studies

**Figure 6:**
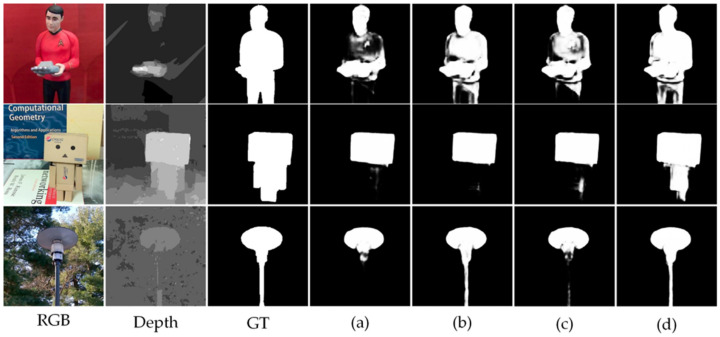
Visual comparisons for showing the effectiveness of different components.

To objectively evaluate the effectiveness of individual modules, we design three variant models (a), (b), (c), and conduct experiments on the challenging DUT-RGBD and SIP datasets. The variant (d) is our full SFCINet, and the results are shown in Table 3 and visualized in Figure 6. Since our proposed module is a unified framework, for example, the global frequency-synergized prior $P_{i}$ is generated by the SFS and flows to the CHD, we cannot simply remove the module. The core of the SFS is to shift the perspective from the spatial domain to the frequency domain, thereby obtaining the global frequency information. For the variant (a), we remove the frequency domain branch in SFS, while the operations in the spatial domain remain. Meanwhile, we exclude the injection of global features in the CHD. For the variant (b), we restore the SFS module, but block the global frequency-synergized prior from flowing to the CHD. For (c), we replace the cross-modal spatial attention interaction mechanism with concatenation, while leaving the other modules unchanged.

(1) The effectiveness of the SFS module

The effectiveness of the SFS is verified by comparing variants (a) and (b). In contrast to the variant (a), (b) has global perception from a frequency-domain perspective, effectively suppressing background noise and capturing global texture information. In all the metrics, (b) is superior to (a). For example, the F-measure is improved from 0.939 to 0.942 on DUT-RGBD. As shown in Figure 6, the variant (a) completely loses the thin pole due to a lack of global context, while (b) successfully captures the whole salient object. These results fully demonstrate the effectiveness of SFS, which combines the spatial-frequency domain and local-global information. Furthermore, we provide a detailed quantitative analysis in Table 4 to demonstrate the superior global modeling capability of our frequency-domain SFS module over conventional lightweight attention mechanisms, as well as the individual contributions of the amplitude and phase branches. Specifically, we construct variant (1) CA + SA, where the frequency domain within SFS is replaced by a standard combination of Channel Attention (CA) and Spatial Attention (SA), while keeping all other architectures identical. Ours comprehensively outperforms it, improving F-measure from 0.902 to 0.911 on SIP, proving that frequency-domain learning captures global context far better than these lightweight attention modules. Additionally, disabling the amplitude branch (2) or phase branch (3) both causes distinct performance drops. Crucially, while individually disabling either branch, variant (2) or (3) yields performance levels roughly similar to or slightly better than the (1), while jointly enabling both (Ours) brings a pronounced performance leap.

(2) The effectiveness of the CMGI module

To evaluate CMGI, we compare (c) with our full SFCINet (d). We find that the performance of (c) deteriorated in all metrics. The representations of depth and RGB features have significant differences, which result from the fundamental difference of the image sensors. For instance, the noise in the depth map can easily contaminate the significant areas. The results prove that our cross-modal spatial attention interaction mechanism effectively alleviates this issue. From Figure 6, we can also clearly see that (d) provides more accurate positioning for salient objects.

(3) The effectiveness of the CHD module

CHD employs the strategy that injects the global signal to participate in decoding, which can be verified by the comparison (b) with (d). It can be clearly seen that our full SFCINet leads comprehensively in evaluation metrics. It strongly explains that there will be a loss of the overall signal due to upsampling during the decoding process. Our unified framework is designed to alleviate this issue, which leverages global frequency-synergized prior from the SFS. Moreover, in Figure 6, we can clearly see that (d) has a more powerful capacity of capturing the human subjects and the main body area of the figurine, showing the effectiveness of our design.

(4) Efficiency Analysis

We further analyze the computational complexity and inference efficiency of our proposed modules, as detailed in Table 5. The inference speed (FPS) is benchmarked on a single NVIDIA GeForce RTX 4060 GPU with an input resolution of 256×256 and a batch size of 1 under FP16 mixed precision. To measure pure forward inference latency after sufficient warm-up, the entire network execution is accelerated via CUDA Graph, while disk I/O and data pre-processing overheads are excluded; (a) provides the lightest architecture with 9.88M parameters and 277 FPS. We achieve a significant increase in performance at a minor cost of 0.2M Params and 0.25G FLOPs, compared to our full method. When comparing several variants together, we will find that the overall design of our module is extremely lightweight, while the parameters and computational requirements derive from the native backbone network. It is well worth mentioning that our full SFCINet (d) achieves the best state-of-the-art accuracy across all datasets while running at a high speed of 236 FPS, verifying its excellent efficiency and potential for practical deployment. This firmly establishes that our architectural design is highly efficient and achieves an optimal trade-off between detection accuracy and deployment efficiency.

### 4.6. Failure Case Analysis

**Figure 7:**
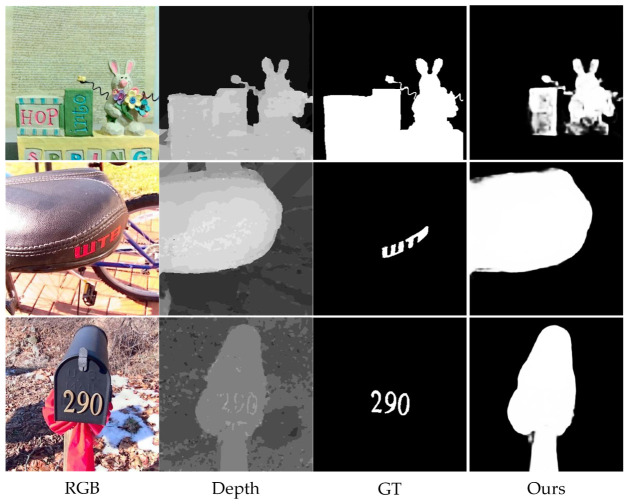
Visual examples of failure case.

As illustrated in Figure 7, our SFCINet encounters performance bottlenecks when dealing with multi-scale semantic ambiguity, specifically where the ground truth (GT) targets are restricted to highly localized, fine-grained textual signs. Depth sensors fail to highlight salient objects on the surface of the macroscopic regions (rows 2 and 3) or in adjacent regions (row 1), as they share the same depth values. Since our SFS module explicitly leverages the amplitude spectrum to capture macro-geometric energy, it understandably prioritizes the holistic structural consistency of the object over pixel-level local areas when facing deceptive depth maps. The subsequent network follows this deceptive consensus, especially the injection of the output prior into the decoding stage, failing to segment localized pixel-level regions. Consequently, dynamically decoupling macro-structural layouts from fine-grained semantic masks remains a vital direction for our future improvement.

## 5. Conclusions

In this paper, we propose a novel and lightweight network for RGB-D salient object detection, termed SFCINet. We employ MobileViT to independently extract multi-scale features. To better capture the global context information and calibrate the features of RGB and depth images, we design the SFS module, which shifts the perspective from the spatial domain to the frequency domain, efficiently perceiving global signals. Given the representation discrepancy between the depth and RGB features, we further develop the CMGI module with a reciprocal anchoring mechanism. It helps select useful information and filter out irrelevant noise, achieving effective integration. During the decoding process, global information is prone to being lost during upsampling. To alleviate this issue, we utilize the global frequency-synergized prior from the SFS module to participate in the decoder, forming the SFCINet into a unified framework. We conduct experiments on five widely accepted datasets and adopt five evaluation metrics to comprehensively verify the outstanding performance of our proposed method. The results show that the SFCINet achieves a desirable trade-off between detection performance and efficiency.

## Figures and Tables

**Table 1:** Results of our model compared with 8 heavyweight methods. The best results are in bold. The symbol “↑” indicates a higher value is better, and “↓” indicates a lower value is better.

Datasets	Metrics	TIP21	TMM22	TIP23	TCSVT23	TOMM23	TIP23	TMM24	NN24	Ours
		CDNet	CCAF	CAVER	MPDNet	AMINet	HiDANet	TPCL	DMGNet	
Params (M)		32.9	41.8	55.8	52.0	199.1	523.0	129.47	-	10.08
FLOPs (G)		72.0	76.6	21.86	52.0	124.7	71.5	212.02	-	4.27
SIP	$F_{\beta }\;↑$	0.805	0.864	0.884	0.884	-	0.893	0.903	0.877	**0.911**
	M ↓	0.076	0.054	0.043	0.048	-	0.043	0.038	0.049	**0.041**
	S ↑	0.823	0.876	0.893	**0.904**	-	0.892	0.900	0.878	0.899
	E ↑	0.880	0.916	0.927	0.925	-	0.930	0.937	0.924	**0.943**
NJU2K	$F_{\beta }\;↑$	0.866	0.897	0.874	0.892	0.909	**0.921**	0.918	0.884	**0.921**
	M ↓	0.048	0.037	0.032	0.041	0.036	**0.029**	0.028	0.035	0.031
	S ↑	0.885	0.910	0.920	0.912	0.906	**0.926**	**0.926**	0.913	0.920
	E ↑	0.908	0.942	0.922	0.937	0.943	**0.952**	0.928	0.930	**0.952**
NLPR	$F_{\beta }\;↑$	0.848	0.881	0.895	0.897	0.893	0.910	0.908	0.893	**0.921**
	M ↓	0.032	0.026	0.022	0.023	0.025	**0.021**	0.017	0.022	0.023
	S ↑	0.902	0.922	0.929	**0.932**	0.907	0.929	0.935	0.924	0.929
	E ↑	0.935	0.953	0.959	0.951	0.950	0.959	**0.965**	0.956	**0.965**
STERE	$F_{\beta }\;↑$	0.873	0.869	0.872	0.884	0.882	0.897	0.896	0.903	**0.907**
	M ↓	0.042	0.044	0.034	0.039	0.040	0.035	0.031	**0.032**	0.037
	S ↑	0.896	0.891	0.914	**0.915**	0.890	0.911	0.916	0.917	0.910
	E ↑	0.922	0.933	0.931	0.936	0.937	0.944	0.928	0.950	**0.951**
DUT-RGBD	$F_{\beta }\;↑$	0.874	0.904	0.919	-	**0.950**	0.920	0.935	-	0.947
	M ↓	0.048	0.038	0.029	-	**0.022**	0.032	0.024	-	0.026
	S ↑	0.880	0.903	0.930	-	**0.944**	0.924	0.936	-	0.935
	E ↑	0.918	0.944	0.955	-	**0.968**	0.951	0.960	-	**0.968**

**Table 2:** Results of our model compared with 8 lightweight methods. The best results are in bold. The symbol “↑” indicates a higher value is better, and “↓” indicates a lower value is better.

Datasets	Metrics	TIP22	TIP22	TIP23	TCSVT24	TCSVT24	KBS24	TMM25	NN25	Ours
		MoADN	MMF	LSNet	AirSOD	HENet	MAGNet	FasterSal	BTNet	
Params (M)		5.0	3.9	5.4	2.4	2.7	5.2	5.2	3.8	10.08
FLOPs (G)		1.58	10.9	1.21	0.9	2.2	2.2	0.9	7.2	4.27
SIP	$F_{\beta }\;↑$	0.850	0.871	0.881	0.855	0.894	0.894	0.870	0.891	**0.911**
	M ↓	0.058	0.048	0.050	0.060	**0.041**	**0.041**	0.049	0.046	**0.041**
	S ↑	0.865	0.882	0.886	0.859	**0.899**	**0.899**	0.870	0.890	**0.899**
	E ↑	0.911	0.919	0.920	0.904	0.933	0.932	0.929	0.923	**0.943**
NJU2K	$F_{\beta }\;↑$	0.892	0.885	0.899	0.889	0.899	0.900	0.906	0.911	**0.921**
	M ↓	0.041	0.042	0.039	0.039	0.034	0.034	0.034	0.035	**0.031**
	S ↑	0.906	0.898	0.911	0.908	0.918	0.918	0.908	**0.925**	0.920
	E ↑	0.935	0.925	0.939	0.934	0.912	0.942	0.949	0.944	**0.952**
NLPR	$F_{\beta }\;↑$	0.875	0.887	0.891	0.884	0.892	0.904	0.902	0.911	**0.921**
	M ↓	0.027	0.027	0.025	0.023	0.025	**0.021**	0.022	**0.021**	0.023
	S ↑	0.915	0.917	0.919	0.925	0.916	**0.932**	0.920	0.927	0.929
	E ↑	0.947	0.943	0.951	0.957	0.958	0.962	0.960	0.956	**0.965**
STERE	$F_{\beta }\;↑$	0.868	0.881	0.850	0.865	0.895	0.895	0.875	0.893	**0.907**
	M ↓	0.042	0.039	0.055	0.043	**0.036**	**0.036**	0.040	0.038	0.037
	S ↑	0.898	0.903	0.871	0.895	**0.916**	**0.916**	0.888	0.920	0.910
	E ↑	0.935	0.936	0.909	0.932	0.933	0.943	0.939	0.938	**0.951**
DUT-RGBD	$F_{\beta }\;↑$	0.923	0.920	0.844	-	0.922	0.921	0.925	0.929	**0.947**
	M ↓	0.031	0.033	0.061	-	0.031	0.030	0.030	0.028	**0.026**
	S ↑	0.927	0.907	0.867	-	0.930	0.929	0.918	0.927	**0.935**
	E ↑	0.959	0.945	0.887	-	0.950	0.955	0.958	0.956	**0.968**

**Table 3:** Quantitative comparisons between our model and different variants. The best is in bold. The symbol “↑” indicates a higher value is better, and “↓” indicates a lower value is better. The symbol “✓” indicates that the corresponding module is retained, while “х” indicates that the module is removed.

Variant	SFS	CMGI	CHD	DUT-RGBD				SIP			
				$F_{\beta }\;↑$	M ↓	S ↑	E ↑	$F_{\beta }\;↑$	M ↓	S ↑	E ↑
(a)	х	✓	х	0.939	0.029	0.929	0.963	0.902	0.046	0.892	0.935
(b)	✓	✓	х	0.942	0.027	0.933	0.965	0.905	0.044	0.893	0.938
(c)	✓	х	✓	0.943	0.028	0.932	0.964	0.902	0.043	0.894	0.936
(d)	✓	✓	✓	**0.947**	**0.026**	**0.935**	**0.967**	**0.911**	**0.041**	**0.899**	**0.943**

**Table 4:** Quantitative comparisons within the SFS. The best is in bold. The symbol “↑” indicates a higher value is better, and “↓” indicates a lower value is better.

Variant	DUT-RGBD				SIP			
	$F_{\beta }\;↑$	M ↓	S ↑	E ↑	$F_{\beta }\;↑$	M ↓	S ↑	E ↑
(1) CA + SA	0.944	0.027	0.932	0.964	0.902	0.043	0.892	0.937
(2) w/o amplitude	0.942	0.029	0.931	0.962	0.906	0.043	0.894	0.939
(c) w/o phase	0.943	0.028	0.933	0.965	0.907	0.045	0.890	0.935
Ours	**0.947**	**0.026**	**0.935**	**0.967**	**0.911**	**0.041**	**0.899**	**0.943**

**Table 5:** Computation complexity and efficiency comparisons between our model and different variants. The symbol “✓” indicates that the corresponding module is retained, while “х” indicates that the module is removed.

Variant	SFS	CMGI	CHD	Params	FLOPS	FPS
(a)	х	✓	х	9.88	4.02	277
(b)	✓	✓	х	10.05	4.24	232
(c)	✓	х	✓	10.07	4.26	238
(d)	✓	✓	✓	10.08	4.27	236

## Data Availability

Data are contained within the article. The code of our proposed SFCINet can be found in https://github.com/luyitong530-cloud/SFCINet.git (accessed on 8 June 2026).
